# Bovine Papillomavirus Genotypic Diversity and a Putative Novel Viral Type in Ecuador

**DOI:** 10.3390/vetsci12070672

**Published:** 2025-07-17

**Authors:** Diego J. Carvajal-Reina, Fausto Bedoya-Páez, Mónica Salomé Guerrero-Freire, Yanua Ledesma, David Vasco-Julio, Jacobus H. de Waard, Armando Reyna-Bello

**Affiliations:** 1 Grupo de Investigación en Sanidad Animal y Humana (GISAH), Departamento de Ciencias de la Vida y la Agricultura, Carrera de Biotecnología, Universidad de las Fuerzas Armadas ESPE, Sangolquí 171103, Ecuador; carvajaldiego025@gmail.com (D.J.C.-R.); fvbedoya@espe.edu.ec (F.B.-P.); 2 Programa de Doctorado, Facultad de Ciencias Veterinarias, Universidad de Buenos Aires, Buenos Aires C1428EGA, Argentina; monica.guerrero.freire@udla.edu.ec (M.S.G.-F.); yanua.ledesma@udla.edu.ec (Y.L.); 3 One Health Research Group, Facultad de Medicina Veterinaria, Universidad de las Américas, Quito 170125, Ecuador; 4 Posgrado en Ciencias Biológicas, Unidad de Posgrado, Edificio D, 1° Piso, Circuito de Posgrados, Ciudad Universitaria, Ciudad de México 04510, Mexico; david.vasco.julio@outlook.com; 5 Centro de Investigación Sobre Enfermedades Infecciosas, Instituto Nacional de Salud Pública, Cuernavaca 62050, Mexico

**Keywords:** bovine papillomavirus (BPV), human papillomavirus (HPV), BPV types, novel virus type, co-infection, benign and malignant neoplasms

## Abstract

Bovine papillomatosis, caused by a growing group of bovine papillomaviruses (BPVs), is a disease with benign proliferative lesions (papillomas) that may progress to malignancies due to immunological, environmental, or viral factors. This study investigated BPV type diversity in cattle from the Province Santo Domingo de Tsáchilas in Ecuador. Warty lesions were collected from 30 cattle across eight farms. Nucleic acids were extracted using a silicon dioxide-based method, and the partial L1 gene was amplified with PCR. DNA sequences were analyzed using maximum likelihood phylogenetics. Fifty-seven warty lesions yielded ten well-known BPV types: BPV1, BPV2, BPV4, BPV6, BPV8, BPV9, BPV10, BPV13, BPV14, and BPV42. Recently described viral types, BPV-CR2 from Costa Rica and BPV/BR-UEL08 from Brazil, were also detected, alongside a putative novel viral type, BPVEC2024-6-22.1—likely belonging to the genus *Xipapillomavirus*. This genus had the highest overall count. In contrast, *Deltapapillomaviruses* were found across all sampled farms. This study underscores BPV diversity in this localized region of Ecuador, and includes genotypes linked to cancers such as enzootic hematuria. The findings provide important epidemiological insights, contributing to vaccine development or immune therapy and improved disease management.

## Simple Summary

Cattle worldwide can develop cauliflower-shaped skin lesions (warts) caused by a virus called bovine papillomavirus or BPV. In this study, warts were collected from 30 cows across eight farms near Santo Domingo, Ecuador. We used a laboratory test (PCR) to detect and analyze the DNA of these viruses, identifying ten BPV types and a possible new, unidentified viral type. These viruses belong to three BPV genera: *Xipapillomavirus*, *Deltapapillomavirus*, and *Epsilonpapillomavirus*. Some of these viruses are linked to more severe diseases, including cancer. In conclusion, we successfully identified specific BPV types present in the country through DNA analysis, which will help to establish control measures and develop new vaccine candidates in the future.

## 1. Introduction

Bovine papillomaviruses (BPVs) are non-enveloped, double-stranded DNA viruses from the family *Papillomaviridae* that infect squamous and mucosal epithelia, causing benign proliferative lesions known as warts or papillomas [1,2,3,4]. The viral genome, 6953–8607 base pairs long, includes early (E1–E7) and late (L1–L2) genes and a non-coding region (LCR/URR) regulating replication and transcription [1,3,5]. These viruses are site- and species-specific and transmitted via direct contact, contaminated surfaces, or flies [6]. BPV infections can compromise cattle health, affecting areas such as the mouth and mammary glands, and are linked to diseases like mastitis, enzootic hematuria, and cancers of the bladder and esophagus, causing mechanical milking difficulties, anemia, and even death [1,4].

BPVs are classified into five genera: *Deltapapillomavirus*, *Xipapillomavirus*, *Epsilonpapillomavirus*, *Dyokappapapillomavirus*, and *Dyoxipapillomavirus*. There are 44 identified types, while viral types 19, 21, and 27 and tick-associated BPV remain unclassified [6,7,8,9,10]. Classification is based on the L1 gene sequence, differing by more than 10% between genotypes [1,2]. Six BPV types (BPV1–6) are most frequently associated with neoplasia; these are well-characterized and cause distinct lesions. BPV1 induces fibro papillomas on teats and skin, BPV2 causes common cauliflower-like warts, and BPV4 is associated with papillomas in the gastrointestinal tract [1,2,4,8]. BPV1 and BPV2 are the most prevalent types and are associated with benign and malignant neoplasms, including bladder cancer and gastrointestinal tumors [1,2,4]. Immunosuppressed animals, especially those exposed to carcinogenic ferns, are at higher risk of malignancies [4]. BPV7-9 have some relevance to benign and potentially malignant lesions, but the evidence is less robust. BPV13 is particularly noteworthy for its potential link to bladder cancer [11]. Other types (BPV10-44) remain under investigation, and their oncogenic potential is largely unknown, though they are typically linked to benign warts [12].

BPV is cosmopolitan and highly prevalent in Asia, Europe, and Africa. In the Americas, several genotypes have been reported in countries such as Mexico, Costa Rica, and Brazil, where approximately 60% of the region’s cattle are raised—a figure that may be underestimated due to asymptomatic infections [1,4,13,14,15]. This global and regional distribution highlights the need for local molecular surveillance, especially in underreported regions like Ecuador. Also, co-infections involving multiple BPV types on the same animal are documented. In Ecuador, BPV types 3, 6, 7, and 9 have been identified by PCR-RFLP methods [4,16]. To date, no molecular studies have provided genotypic resolution or sequence-based characterization in the country. This study presents the first molecular and phylogenetic characterization of BPV genotypes in Ecuador, based on samples collected from cattle in farms located in and around the Santo Domingo de los Tsáchilas Province, using PCR-sequencing and bioinformatic analyses. Our findings aim to unravel BPV genetic diversity in local herds, inform control strategies, and support future vaccine development.

## 2. Materials and Methods

### 2.1. Sampling

**Figure 1:**
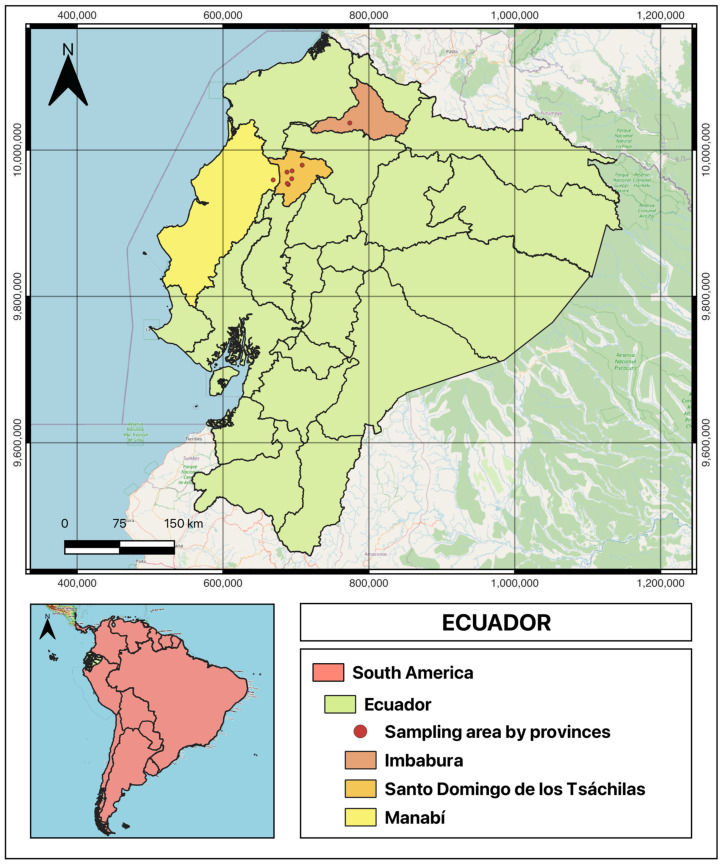
Geographic location of sampled farms in Ecuador. The map highlights Ecuador within South America and shows the provinces of Santo Domingo de los Tsáchilas, Manabí, and Imbabura. Sampled farm locations are indicated. The map was created using QGIS (version 3.42).

Sampling was conducted from September to December 2023, in the humid tropical province of Santo Domingo de los Tsáchilas, characterized by an average temperature of 22.9 °C, annual precipitation of 3000–4000 mm, and an altitude of 655 m. Additionally, one farm each in the provinces of Manabí and Imbabura was visited for sampling (see map in Figure 1). Due to the logistical limitations of accessing rural farms and the need to identify animals with visible lesions opportunistically, a non-probabilistic convenience sampling strategy was employed to identify and characterize BPVs in the area using the “snowball” method [15], whereby cattle with lesions suggestive of papillomatosis were identified through farm referrals. After informal interviews with farm personnel and signing an informed consent, suspected animals were inspected in holding pens. Data collected included animal identification, location, size, and macroscopic morphology. No histological analysis was performed to confirm tissue composition. Photographs were taken prior to excision, and lesions were removed with sterilized forceps and surgical scissors. Samples were immediately placed in sterile 15 mL tubes with 70% ethanol, labeled with farm and sample identifiers, and stored in a cooler at 4 °C. In some cases, 2–3 separated lesions were collected from different anatomical sites on the same animal. After sampling, antiseptics were applied to the excision site to prevent infection, and instruments were disinfected with 70% ethanol between animals. All samples were transported to the Molecular Biology laboratory at UFA-ESPE University in Santo Domingo, where they were stored at −20 °C.

Sampling took place on eight farms: seven in Santo Domingo de los Tsáchilas (three in the “El Carmen” sector, three in the “Luz de América” parish, and one in the “Libertad del Toachi” parish, located in Manabí Province) and one farm in Ibarra, Imbabura Province. From farms 1–8, respectively, 2, 12, 18, 2, 3, 5, 3, and 12 samples were collected, yielding a total of 57 warty lesions. See Figure 1 for the geographic location of the sampled farms.

### 2.2. DNA Extraction

Using sterilized forceps and scissors, an approximately 10 mg piece of the excised warty lesion was cut and placed in a 1.5 mL tube. The ethanol was evaporated at 37 °C for 30 min. Then, 370 µL of Tris-HCl buffer (10 mM, pH 8.0) was added, and the samples were homogenized. Afterward, 30 µL of proteinase K (20 mg/µL, Promega, Madison, WA, USA) was added, and the tubes were vortexed and incubated in a water bath at 56 °C for 24 h.

Next, 200 µL of Glassmilk (guanidine-HCl 6 M, urea 10 mM, Tris-HCl 10 mM, Triton-X 20%, silicon dioxide 40mg/mL) was added, and the mixture was vortexed. DNA was isolated as previously described [17]. The DNA–silicon dioxide pellet was resuspended in 200 µL of TE buffer (10 mM Tris-HCl, pH 8.0, 1 mM EDTA), stored at 4 °C for 24 h, and then kept at −20 °C for further analysis. DNA concentration was measured using a Nanodrop Lite spectrophotometer (Thermo Scientific, Waltham, MA, USA), and DNA integrity was assessed by agarose gel electrophoresis.

### 2.3. Identification of Bovine Papillomavirus: Amplification and Sequencing

Bovine papillomavirus identification and molecular characterization were performed using PCR sequencing as described by Forslund et al. with the following degenerate primers: FAP59 (forward), 5′-TAACWGTIGGICAYCCWTATT-3′, and FAP64 (reverse), 5′-CCWATATCWVHCATITCICCATC-3′ [18]. A PCR protocol was optimized for partial amplification of the BPV L1 gene, based on the original cycling conditions reported by Forslund et al. [18], with modifications established through standardization in our laboratory. A temperature gradient (42–50 °C) was applied during optimization to determine the ideal annealing temperature. The final amplification program consisted of an initial denaturation at 94 °C for 10 min, followed by 32 cycles of 30 s at 95 °C, 45 s at 48 °C, 80 s at 72 °C, with a final extension at 72 °C for 10 min. PCR reactions were carried out using Taq Polymerase (abm, Richmond, BC, Canada), following the manufacturer’s instructions. Approximately 50 ng of DNA was used for each reaction. Each PCR run included a negative control (nuclease-free distilled water) to monitor contamination and a previously validated positive control to confirm amplification performance. PCR products were visualized via agarose gel electrophoresis and subsequently sequenced using Sanger sequencing with an ABI 3500xL Genetic Analyzer (Applied Biosystems, Waltham, MA, USA) in a BigDye 3.1^®^ (Thermo Scientific, Waltham, MA, USA) capillary electrophoresis matrix.

### 2.4. Phylogenetic and Data Analysis

The resulting sequences were analyzed using bioinformatics tools to construct a phylogenetic tree based on the maximum likelihood method with rapid bootstrapping. Sequences were manually reviewed and quality-checked using Pytho (version 3.11) and the Pandas package (version 2.3.1). Each chromatogram was verified to ensure accuracy in base calling, and all accepted sequences had a Phred quality score above 20. All sequences were cleaned in MEGA11 (version 0.1) and compared using BLAST against the NCBI database (available at: https://blast.ncbi.nlm.nih.gov/Blast.cgi, accessed on 13 March 2023) and the PaVe papilloma genomes database (available at: https://pave.niaid.nih.gov/search/pv_specific_blast, accessed on 1 July 2023). Reference sequences with a higher identity in BLAST were selected, along with papillomavirus sequences from different species as outgroups. These sequences were aligned in MEGA11 using the CLUSTAL W algorithm and refined with GBLOCKS 0.91b. Then, a phylogenetic tree was inferred in MEGA11 using maximum likelihood, the Kimura 2-parameter model, and 1000 bootstrap replicates. The final dataset included 43 nucleotide sequences with 469 positions.

Descriptive statistics were used to summarize the frequency and anatomical distribution of the identified BPV types. Graphs showing the relative and absolute abundance of BPV genotypes were generated using R software (version 4.2.3). No inferential statistical tests were applied, as the study employed non-probabilistic convenience sampling and was not designed to support comparisons between groups or farms.

### 2.5. Ethics Approval and Consent to Participate

This investigation was approved and financed by the Research Committee of the Faculty of Veterinary Medicine at the Universidad de Las Americas (VET-22.01). For that, our research adhered to internationally recognized ethical standards, including the European Convention for the Protection of Vertebrate Animals Used for Experimental and Other Scientific Purposes (Strasbourg Convention). We followed the 3Rs principle (Replace, Reduce, Refine) to ensure humane treatment of bovines, minimizing pain, suffering, and distress. The study was approved, and informed consent was obtained from farm owners. All personnel were trained in animal welfare and handling practices consistent with the convention’s guidelines. All samples were collected under veterinary supervision using superficial and minimally invasive methods. Following collection, the sampling sites were sprayed with antiseptics to prevent infection or myiasis.

## 3. Results

### 3.1. Samples

**Figure 2:**
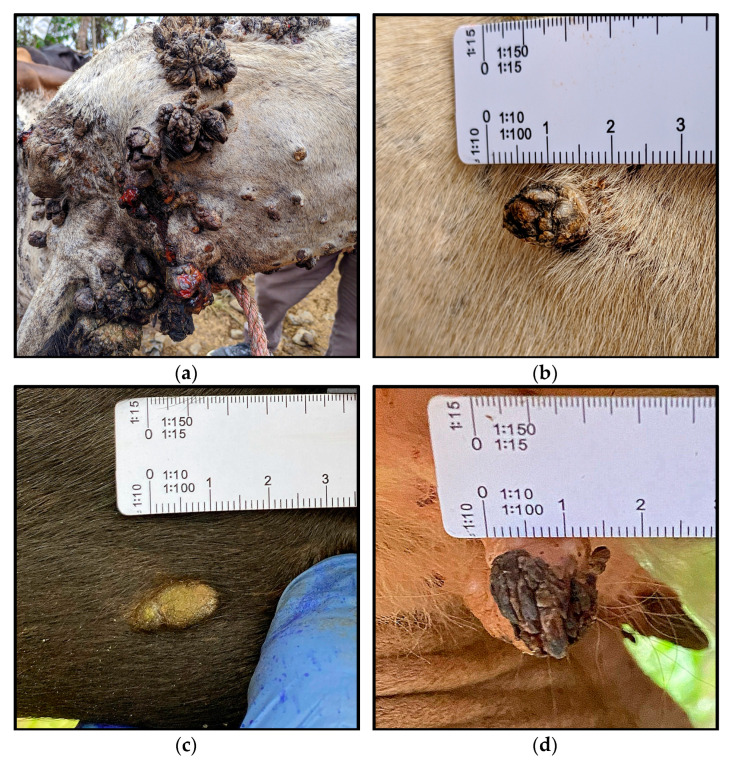

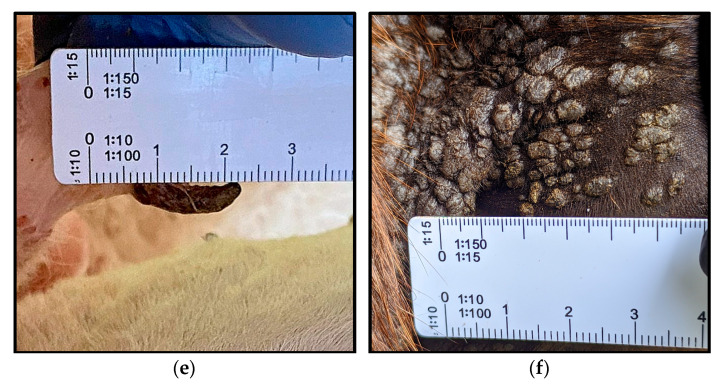
Photographic report and observed morphology of warty skin lesions. (**a**) A calf with generalized papillomatosis showing multiple warty lesions, infected with the BPV13 viral type; lesions measured approximately 4–5 cm in diameter at the time of inspection, primarily on the face and neck. (**b**) Sample F205_310823 (BPV13, cauliflower shape, extracted from the neck). (**c**) Sample F315_061023 (BPV6, rice shape, extracted from the neck). (**d**) Sample F313_061023 (BPV6, filamentous, extracted from the udder). (**e**) Sample F316_061023 (BPV6, pedunculated, removed from the udder). Sample (**f**) F829_091223 (BPV8, flattened, removed from the tail). A millimeter ruler is included for scale.

A total of 57 warty skin lesions were collected from 30 cattle across eight farms. Of these, one lesion was collected from 9 cattle, two from 15, and three from 6 cattle. Most samples were taken from the neck (23.6%), followed by the udder and ear (12.7% each), leg (10.9%), navel (7.3%), head, face, and back (5.5% each), mouth (3.6%), and tail and nose (1.8%). The average size of the lesions was 0.8 cm. Among the 57 lesions, the most frequent macroscopic morphology was cauliflower-shaped, followed by rice-like, filamentous, flattened, and pedunculated forms (Figure 2).

### 3.2. Amplification, Sequencing and Phylogeny

Of the 57 DNA samples, 49 tested positive for bovine papillomavirus, showing PCR amplicons of approximately 500 bp on agarose gel electrophoresis. Sanger sequencing produced 47 high quality electropherograms and 2 with background noise. BLAST analysis of the sequences obtained using the forward primer (FAP59) revealed 13 distinct BPV types. For representative samples, reverse primer sequencing was also performed, and alignment with the FAP59 sequences allowed the determination of partial L1 gene fragments ranging from 425 to 433 bp. All partial sequences were deposited in GenBank with their corresponding accession numbers, as listed in Table 1.

Ten of these thirteen sequences had a high percentage of identity (99–100%) with the viral types BPV1, BPV2, BPV4, BPV6, BPV8, BPV9, BPV10, BPV13, BPV14, and BPV42; two of them (isolates BPV-EC2024-3-17 and BPVEC2024-3-9) showed high identity with recently characterized new types of BPVs from Costa Rica (BPV-CR2. MZ292467.1) and Brazil (BPV-BR-UEL08. MH729203.1) [15,19], respectively. Finally, BPVEC2024-6-22.1 (PV032937) showed 81.71% identity with the closest reference sequences in the NCBI database and 77.88% in the PaVe database. Since papillomavirus types are considered novel when they differ by more than 10% in the L1 gene sequence [1,2], this sequence meets the criteria for classification as a putative novel BPV type (Table 1). Whole genome sequencing of the complete genome of this putative new papillomavirus type is required to confirm this.

**Figure 3:**
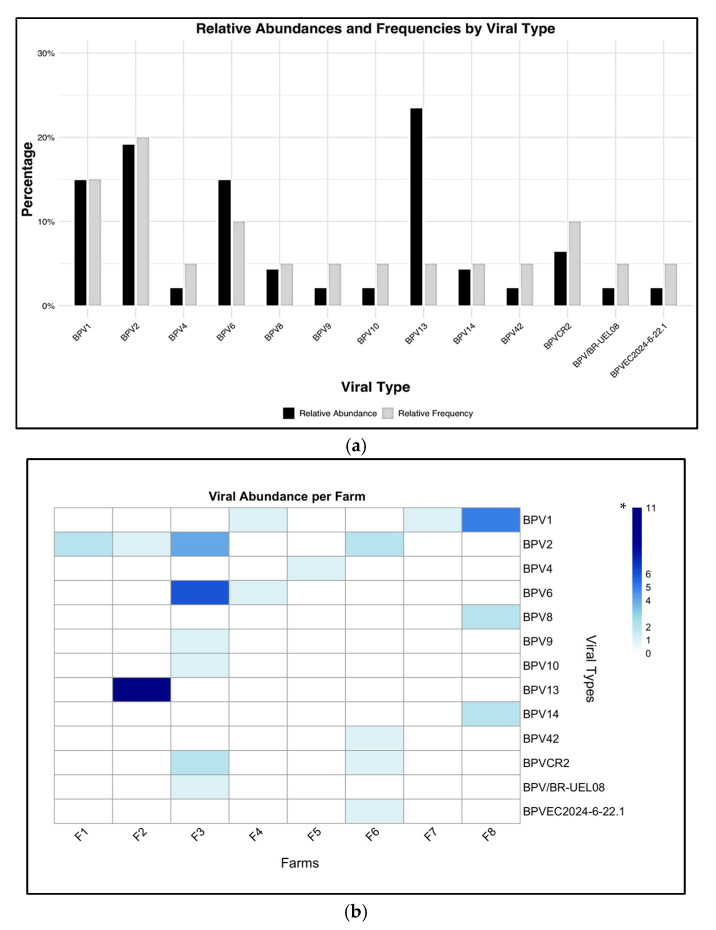
Diversity of identified BPV types: frequency and abundance. (**a**) Graph showing the relative abundance and frequency of BPV genotypes. (**b**) Graph displaying the absolute abundance of BPV genotypes, grouped by farm. * Number of times the viral type was detected per farm. Graphs were generated using R software (version 4.2.3).

Regarding the distribution of BPV on the eight farms, it was observed that the most frequent genotype was BPV2, found in four of the eight farms and representing 19% of all samples, as shown in Figure 3. Although BPV13 presented the highest rate (23%), it was geographically limited only to farm 2. The next most frequent genotypes were BPV1, distributed on farms 4, 7, and 8, and BPV6, distributed on farms 3 and 4, respectively. On the other hand, Farm 3, located on the outskirts of the Luz de América parish, was the one with the greatest genotypic diversity with six different types identified (BPV6, BPV2, BPV10, BPV9, BPV-CR2, and BPV-BR-UEL08) followed by farm 6 with three different types (BPV2, BPV42, and BPV-CR2) and the new putative type of BPV (BPVEC2024-6-22.1). Finally, the types with the lowest incidence (BPV8, BPV14, BPV4, BPV9, BPV10, and BPV42) were distributed on farms 3, 5, 6, and 8.

Co-infections were observed in seven cattle (Table 2). BPV13 and BPV2 were detected in different warty lesions located on the neck of the same bovine. BPV42, BPV-CR2, and the putative new type BPVEC2024-6-22.1 were found in a single bovine in lesions located on the nose, neck, and back, respectively. Similarly, BPV14, BPV1, and BPV2 were identified in another bovine in lesions on the back and neck. On farm 3, several co-infections were recorded: BPV2 and BPV9 were detected in one individual, in lesions on the mouth and navel, respectively. In another case, BPV2, BPV6, and BPV10 were found in a single animal in the chest, ear, and udder, respectively. Notably, the recently described BPV-CR2 and BPV-BR-UEL08 were detected together in the ear and udder of the same animal.

On the same farm, two potential co-infections involving multiple BPV genotypes were observed in the same lesion, one located on the ear of one bovine and the other on the udder of another. The corresponding sequencing electropherograms showed excessive background noise. This phenomenon is likely attributed to the presence of co-infections [20].

**Figure 4:**
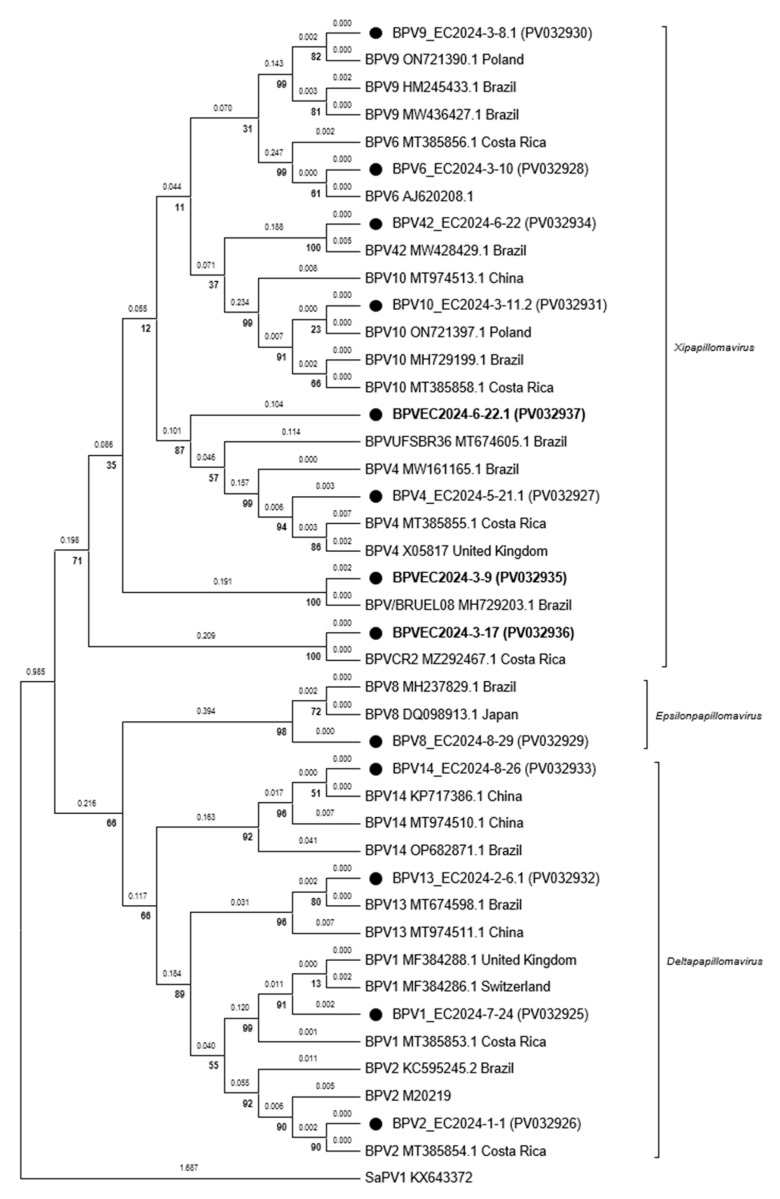
Phylogenetic analysis carried out with the types of BPVs found in this study. The phylogenetic tree was constructed with MEGA11 using maximum likelihood, the Kimura 2-parameter model, and 1000 bootstrap replicates. The viral types found in this study are identified with a black dot. The bolded entries represent viral isolates that currently do not have an alphanumeric type code assigned, according to the PaVe database.

Finally, regarding phylogenetic analysis (Figure 4), we observed a distribution of the genotypes in three genera: *Xipapillomavirus*, *Deltapapillomavirus*, and *Epsilonpapillomavirus*. Within each genus, the distribution by pathotype was observed with high bootstrap values for each type. Likewise, it is important to note that the putative novel type of BPV (BPVEC2024-6-22.1) clustered within the genus *Xipapillomavirus*, sharing a common ancestor with BPV4.

## 4. Discussion

In this study, bovine warty skin lesions were collected from farms around Santo Domingo de los Tsáchilas Province in Ecuador. Lesions suggestive of papillomatosis were sampled from various anatomical locations in affected cattle, with multiple lesions sometimes taken from the same animal. The most common morphology observed was the cauliflower-like structure (35.1%), consistent with previous reports describing pedunculated, dry, and warty appearances in cattle [2,8]. The absence of BPV DNA in eight samples may be attributed to factors such as low viral load, DNA degradation, or the presence of other skin conditions that mimic papillomatosis. As no histological analysis was performed to confirm tissue composition, we cannot exclude the possibility that these lesions were non-papillomatous.

This study marks the first genotypic characterization of bovine papillomaviruses (BPVs) in Ecuador using PCR and sequencing approaches. Thirteen BPV genotypes were identified across three Ecuadorian provinces, including 10 matching known types (99–100% identity) from the genera *Xipapillomavirus* (BPV4, BPV6, BPV9, BPV10, and BPV42), *Deltapapillomavirus* (BPV1, BPV2, BPV13, and BPV14), and *Epsilonpapillomavirus* (BPV8). Two additional types, (BPVEC2024-3-9 and BPVEC2024-3-17), both from the genus *Xipapillomavirus*, corresponded to recently reported viruses from Brazil (BPV-BR-UEL08 (MH729203.1)) and Costa Rica (BPV-CR2 (MZ292467.1)) [15,19]. Finally, BPVEC2024-6-22.1 fulfilled the identity thresholds used to define a putative novel viral type, as mentioned before (81.71% identity in NCBI, 77.88% in PaVe). However, its official classification requires further genomic characterization and formal approval by the ICTV.

The genus *Deltapapillomavirus* was the most frequently detected, with BPV2 predominating (19%) and found in diverse anatomical locations, followed by BPV1 (15%). BPV13, although restricted to one single farm, showed the highest occurrence on multiple body parts. BPV6 and other xipapillomaviruses, commonly associated with udder lesions, were also notable due to their reported association with mastitis risks. Co-infections, which have been widely reported in different investigations [2,21], were observed in six animals, including new combinations (e.g., BPV14 + BPV1 + BPV2), underscoring the possible biological relevance of multiple BPV genotypes within the same host.

This study used FAP consensus primers—a pair of degenerate PCR primers—for BPV detection. This is a widely adopted method in BPV research, particularly in studies aimed at identifying different BPV types. However, we acknowledge that the use of a single primer set may limit the detection of multiple BPV types within the same lesion, potentially leading to an underestimation of co-infections. Given these methodological constraints, the BPV types identified in this study likely represent those most efficiently amplified by the FAP primers, rather than a comprehensive profile of all types present in the lesions. Consequently, the relative frequencies reported (Figure 3) should be interpreted with caution, as they reflect only the detectable BPV types rather than their true prevalence. Future studies incorporating type-specific primers could provide a more complete understanding of BPV and their epidemiological relevance.

While no malignant neoplasms were observed in the co-infection cases identified in this study, it is important to consider that co-infections may contribute to neoplastic transformations and the development of papillomatosis [12]. This is especially relevant given the established role of co-infections in human papillomavirus (HPV) in promoting carcinogenesis [22,23,24]. These findings highlight the need for further studies on bovine papillomavirus (BPV) to better understand its oncogenic potential and to draw parallels with HPV-related oncogenesis. Although co-infections were identified in several animals, the detection method used may limit the full resolution of multiple infections. Therefore, these results should be interpreted as preliminary, and future studies using more sensitive approaches are recommended to confirm and further characterize BPV co-infections in Ecuador. Our findings also support implementing precautionary measures—such as reducing fern exposure in grazing areas—to help prevent the progression of papillomas into malignancies.

## 5. Conclusions

This study provides the first molecular characterization of bovine papillomavirus (BPV) genotypes in Ecuador through PCR-based sequencing and phylogenetic analysis, reporting partial L1 gene sequences for 13 BPV types. The analysis revealed the presence of the genera *Xipapillomavirus*, *Deltapapillomavirus*, and *Epsilonpapillomavirus*, with deltapapillomaviruses being the most widely distributed and xipapillomaviruses displaying the highest genotypic diversity. Notably, two BPV types recently reported in 2024, in Brazil and Costa Rica (BPV-BR-UEL08 and BPV-CR2), were detected in our study, along with a putative novel species (BPVEC2024-6-22.1) that clustered within the genus *Xipapillomavirus*. While most warty lesions were benign, viral types such as BPV types 1, 2, 6, 13, and 14—previously associated with conditions like urinary bladder cancer, mastitis, and gastrointestinal tumors—were also identified. Co-infections, involving both novel and known viral types, were observed, including instances of multiple infections within single lesions. This study highlights the genotypic diversity of BPV in Ecuador, and emphasizes the importance of continued surveillance and full-genome sequencing to better understand viral evolution, dispersion, and pathogenic potential. This work lays the groundwork for identifying regionally prevalent viral types in the country and advances efforts toward vaccine development to combat bovine papillomatosis.

## Figures and Tables

**Table 1:** BPV types identified in this study.

Isolate	GenBank Accession Number	Genus	Reference Viral Type			
			NCBI	Id (%)	PaVe	Id (%)
BPV1_EC2024-7-24 sample F724_241123	PV032925	Delta	BPV1 (MT385853.1)	100	BPV1 (X02346)	99.3
BPV2_EC2024-1-1 sample F101_250823	PV032926	Delta	BPV2 (LC426022.1)	100	BPV2 (M20219)	99.3
BPV4_EC2024-5-21.1 sample F521.1_211123	PV032927	Xi	BPV4 (OP682875.1)	99.29	BPV4 (X05817)	99.3
BPV6_EC2024-3-10 sample F310_061023	PV032928	Xi	BPV6 (MH729201.1)	100	BPV6 (AB331651)	100
BPV8_EC2024-8-29 sample F829_091223	PV032929	Epsilon	BPV8 (MH237829.1)	99.75	BPV8 (DQ098913)	99.8
BPV9_EC2024-3-8.1 sample F308.1_061023	PV032930	Xi	BPV9 (ON721390.1)	100	BPV9 (AB331650)	99.1
BPV10_EC2024-3-11.2 sample F311.2_061023	PV032931	Xi	BPV10 (ON721397.1)	100	BPV10 (AB331651)	99.8
BPV13_EC2024-2-6.1 sample F206.1_310823	PV032932	Delta	BPV13 (MG818475.1)	100	BPV13 (JQ798171)	100
BPV14_EC2024-8-26 sample F826_261123	PV032933	Delta	BPV14 (KR868228.1)	99.77	BPV14 (KP276343)	99.8
BPV42_EC2024-6-22 sample F622_241123	PV032934	Xi	BPV42 (MW428429.1)	99.54	BPV43 (MW428429.1)	99.5
BPVEC2024-3-9 sample F309_061023	PV032935	Xi	BPV-BR-UEL08 (MH729203.1)	99.77	BPV40 (MW428425.1)	78.27
BPVEC2024-3-17 sample F317_061023	PV032936	Xi	BPV-CR2 (MZ292467.1)	100	BVP12 (JF834523)	86.4
BPVEC2024-6-22.1 sample F622.1_241123	PV032937	Xi	BPV-UFSBR36 (MT674605.1)	81.71	BPV4 (X05817.1)	77.88

The BPV sequences generated in this study were deposited in GenBank with their respective accession numbers. These sequences were analyzed using BLAST searches in NCBI and the PaVe papillomavirus genome database. The similarity (Id) of the generated sequences to those in these databases is presented. The genus of each BPV type is indicated using the Greek letter abbreviation (e.g., “Xi” for *Xipapillomavirus*, “Delta” for *Deltapapillomavirus*, “Epsilon” for *Epsilonpapillomavirus*).

**Table 2:** Co-infections of different BPVs types on the same bovine.

Farm	Bovine	Sample’s Code	PaVe	Body’s Part
2	04	F204_310823	BPV2	Neck
		F204.1_310823	BPV13	Neck
3	08	F308_061023	*	Ear
		F308.1_061023	BPV9	Mouth
		F308.2_061023	BPV2	Navel
3	09	F309_061023	BPV-BR-UEL08	Ear
		F309.1_061023	BPV_CR2	Udder
3	11	F311_061023	ctioBPV2	Chest
		F311.1_061023	BPV6	Ear
		F311.2_061023	BPV10	Udder
3	12	F312_061023	BPV2	Udder
		F312.1_061023	*	Udder
6	22	F622_241123	BPV42	Nose
		F622.1_241123	BPVEC2024-6-22.1	Neck
		F622.2_241123	BPV_CR2	Back
8	26	F826_261123	BPV14	Back
		F826.1_261123	BPV1	Neck
		F826.2_261123	BPV2	Neck

Seven bovines presented co-infections with different BPV types in warty lesions from different parts of the body. * Samples with background noise in the electropherogram, indicating mixed infection.

## Data Availability

The raw data that support the findings of this article will be made available by the corresponding authors on request.
